# Systematic review and network meta-analysis of robot-assisted gait training on lower limb function in patients with cerebral palsy

**DOI:** 10.1007/s10072-023-06964-w

**Published:** 2023-07-26

**Authors:** Yueying Wang, Peipei Zhang, Chao Li

**Affiliations:** 1 College of Rehabilitation Medicine, Shandong University of Traditional Chinese Medicine, Jinan, China; 2 Department of Rehabilitation Medicine, The Second Affiliated Hospital of Shandong University of Traditional Chinese Medicine, Jinan, China; 3 Department of Rehabilitation and Physiotherapy, The Affiliated Hospital of Shandong University of Traditional Chinese Medicine, Jinan, China

**Keywords:** Robot-assisted walking training, Cerebral palsy, Lower limb function, Systematic review, Network meta-analysis

## Abstract

**Objective:**

This study aimed to evaluate the effectiveness of robot-assisted gait training (RAGT) in treating lower extremity function in patients with cerebral palsy (CP) and compare the efficacy differences between different robotic systems.

**Methods:**

PubMed, Web of Science, Cochrane Library, Embase, CNKI, VIP, CBM, and Wanfang databases were searched to collect randomized controlled trials of RAGT for lower extremity dysfunction in patients with CP from the time the databases were created until December 26, 2022. The D and E of Gross Motor Function Measure-88 (GMFM-88) assessed lower limb motor function. Berg Balance Scale (BBS) was used to assess balance function. Walking endurance and speed were assessed using the 6-minute walk test (6MWT) and walking speed. The modified Ashworth Scale (MAS) was used to assess the degree of muscle spasticity in the lower extremities. The Cochrane Risk Assessment Scale and the Physiotherapy Evidence Database (PEDro) scale were used for qualitative assessment in the studies included. RevMan 5.4 was used for data merging and statistical analysis. R 4.2.0 and ADDIS 1.16.8 were used to map the network relationships and to perform the network meta-analysis.

**Results:**

A total of 14 studies were included in the review. The meta-analysis showed that RAGT significantly improved GMFM-88 D and E, BBS, and 6MWT scores in CP patients compared with conventional rehabilitation. However, for walking speed and MAS, the intervention effect of RAGT was insignificant. The network meta-analysis showed that the best probability ranking for the effect of the 3 different robots on the GMFM-88 D score was LokoHelp (*P* = 0.66) > Lokomat (*P* = 0.28) > 3DCaLT (*P* = 0.06) and the best probability ranking for the GMFM-88 E score was LokoHelp (*P* = 0.63) > 3DCaLT (*P* = 0.21) > Lokomat (*P* = 0.16).

**Conclusion:**

RAGT positively affects walking and balance function in patients with CP, while efficacy in improving gait speed and muscle spasticity is unknown. The best treatment among the different robots is LokoHelp. Future high-quality, long-term follow-up studies are needed to explore the clinical efficacy of RAGT in depth.

## Introduction

Cerebral palsy (CP) is a neurodevelopmental disorder caused by damage to the brain during early development and is characterized clinically by postural and motor dysfunction [1]. Motor dysfunction due to CP is often accompanied by sensory, perceptual, cognitive, communication, and behavioral deficits, as well as epilepsy and secondary musculoskeletal problems [2]. The different clinical features of CP can be divided into spastic, irregular, ataxic, and mixed CP [3]. The prevalence of CP is between 0.20 and 0.35% of surviving infants, and the number of people with CP is currently 17 million worldwide [4]. About 40% of these patients cannot walk independently, which seriously affects their activities of daily living [5]. Clinical rehabilitation interventions for CP patients mainly include neurodevelopmental therapy, physiotherapy, and hydrotherapy, which have achieved some success but are less effective in treating walking and balance functions [6].

Robot-assisted gait training (RAGT) is a new rehabilitation intervention that facilitates repetitive and efficient walking training with external mechanical assistance [7–9]. Lerner found that a robotic exoskeleton improved knee range of motion in patients with spastic CP [10]. Borggraefe trained 20 children with CP on a robot-assisted treadmill for 3 weeks, and they showed significant improvements in standing and walking function [11]. Digiacomo showed that RAGT combined with conventional treatment improved motor performance and endurance in children with CP [12].

However, the number of studies is small, outcome indicators and treatment effects vary, clinical effectiveness remains controversial, and there needs to be more high-quality evidence of evidence-based medicine to support this [13, 14]. Network meta-analysis is a statistical method that quantifies several interventions and prioritizes interventions’ effects based on different outcome indicators. In this study, we used the network meta-analysis method to search randomized controlled trials (RCTs) of RAGT for CP patients, extract relevant outcome indicators to evaluate their efficacy, and compare the differences in the efficacy of different robotic devices to provide evidence-based references for the clinical application of RAGT training.

## Methods

This study was registered on the international system evaluation registration platform PROSPERO (CRD42022366471).

### Search strategy

PubMed, Web of Science, Cochrane Library, Embase, CNKI, VIP, CBM, and Wanfang databases were searched by two researchers using medical mesh words and free words from the establishment of the database to December 26, 2022. The search formula is as follows: #1 “robot-assisted gait training” [MeSH] OR robot OR robotics OR RAGT, #2 “cerebral pals*” [MeSH] OR spastic quadriplegia OR spastic diplegia OR spastic hemiplegia OR CP, #3, #1, and #2. The condition was limited to a randomized controlled trial. Cross-check the results after the search, and discuss the decision with the third researcher in case of disagreement.

### Inclusion criteria

The following are the inclusion criteria:

Study design—Chinese and English RCTs.

Participants—children aged <18 years with a precise diagnosis of CP, regardless of gender and race; the child is conscious, has a reasonable level of intelligence, and can cooperate with treatment and follow-up.

Intervention and control measures—the intervention group received RAGT combined with conventional rehabilitation, while the control group received conventional rehabilitation.

Outcome indicators—the primary outcome measures were Gross Motor Function Measure-88 (GMFM-88) D (standing and standing) and E (walking and running and jumping). The secondary outcome measures were the Berg Balance Scale (BBS), 6-minute walk test (6MWT), walking speed, and Modified Ashworth Scale (MAS).

Data—the experimental data are complete and provide sample sizes, means, and standard deviations within groups.

### Exclusion criteria

Exclusion criteria are as follows: literature from non-randomized controlled trials; animal studies; conference abstracts, duplicate studies; incomplete outcome data; selective reporting studies; unavailable full text; literature not in Chinese or English.

### Study selection and data extraction

Two researchers carried out the search, screening, and collation of the literature and the extraction of data and information independently, and the results were cross-checked after completion. In case of disagreement, a third researcher was assigned to decide. Data extraction included the first author, year of publication, basic information about the study population (sample size, age), basic information about the interventions (interventions, duration of treatment, follow-up, type of robot), and outcome indicators.

### Quality assessment

Two researchers assessed the risk of bias in the included literature according to the Cochrane Risk Assessment Scale and the Physiotherapy Evidence Database (PEDro) scale. The third researcher discussed the decision in any disagreement between the two researchers.

### Statistical analysis

RevMan 5.4 was used for meta-analysis. The data types of the outcome indicators for this meta-analysis were all measures. The standard mean difference (SMD) and its 95% confidence interval (CI) were used for statistical analysis of effect values. A fixed effect model was used to analyze if *I*^2^ ≤ 50% and *P* ≥ 0.1, indicating a small heterogeneity among the included studies. A random effect model was used for analysis if *I*^2^ > 50%, *P* < 0.1, indicating more significant heterogeneity between studies. Subgroup analysis or sensitivity analysis was used to identify sources of heterogeneity. The significance level α=0.05.

R 4.2.0 was used to make the network relationship map of the efficacy comparison of different robotic devices. ADDIS 1.16.8 was used for network meta-analysis. Since there is no closed loop in this study’s evidence relationship network graph, a consistency model was fitted to perform a probabilistic analysis of the outcome indicators. Four Markov chains were set, the initial value of the model was 2.5, the iteration step was fine-tuned by 10, and the number of iterations was 50,000, of which the first 20,000 were annealed to eliminate the effect of the initial value. The last 30,000 were used for sampling. The potential scale reduction factor (PSRF) was used to evaluate the convergence efficiency. The PSRF tends to be 1, indicating good convergence efficiency and high reliability of the network meta-analysis results. The rank-ranking probability diagram presented the probability of each intervention becoming the best intervention, and rank 1 was the best probability. The ranking of interventions’ superiority was based on the magnitude of the rank 1 value. The funnel plot was drawn using RevMan 5.4, and if the funnel plot was symmetrical, it suggested that the possibility of publication bias was low.

## Results

### Study selection and characteristics

**Fig. 1:**
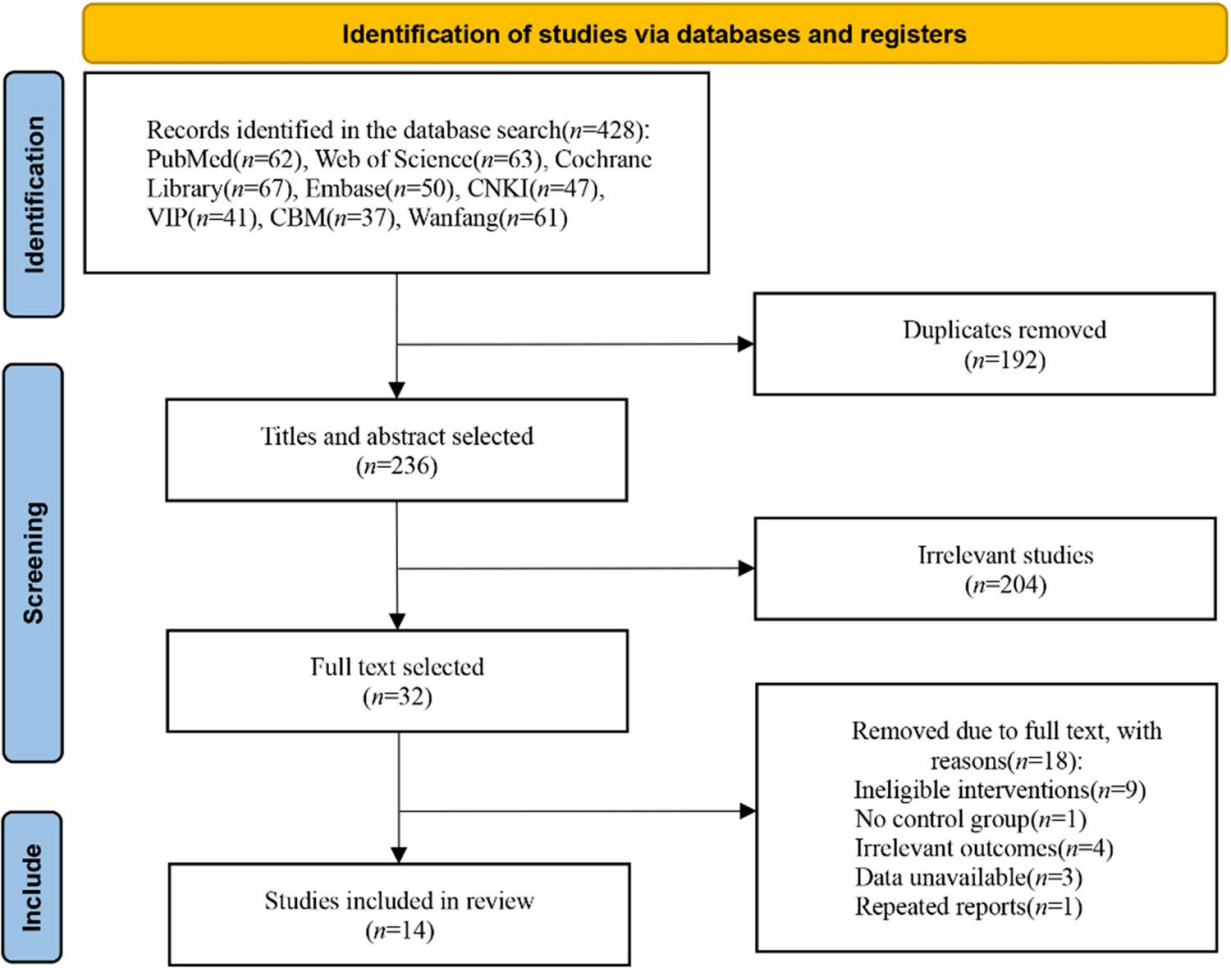
Screening process of literature selection

According to the literature search strategy, 428 studies were obtained from the initial search. After excluding 192 duplicate publications, the remaining 236; after reading the titles and abstracts, 204 were excluded, and the remaining 32. After carefully reading the complete text, 14 studies were finally included, including 8 in Chinese [15–22] and 6 in English [23–28], with a total of 654 patients. The process of literature screening is shown in Fig. 1. The essential characteristics of the included studies are shown in Table 1.

**Table 1:** Characteristics of included studies

Study	Sample (E/C)	Age (year, E/C)	Intervention		Time, frequency	Duration of treatment	Follow-up	Robot	Outcomes
			E	C					
Jin (2012)	16/16	(5.35±0.43)/(5.02±0.78)	RAGT+ conventional rehabilitation	Conventional rehabilitation	20min/day, 6days/week	8 weeks	–	LokoHelp	GMFM-88 D, E
Zhu (2016)	18/18	(5–16)/(5–16)	RAGT+ conventional rehabilitation	Conventional rehabilitation	20min/day, 6days/week	8 weeks	–	LokoHelp	GMFM-88 D, E
Lv (2017)	47/47	6.25/6.27	RAGT+ conventional rehabilitation	Conventional rehabilitation	30min/day	3 months	–	LokoHelp	GMFM-88 D, E; BBS
Yin (2017)	12/11	(29.5±7.1)/(28.2±6.8)	RAGT+ conventional rehabilitation	Conventional rehabilitation	30min/day, 5days/week	8 weeks	–	LokoHelp	BBS; Walking speed; MAS
Zhang (2018)	55/55	(5.42±1.57)/(5.13±1.17)	RAGT+ conventional rehabilitation	Conventional rehabilitation	30-40min/day, 5days/week	8 weeks	–	LokoHelp	GMFM-88 D, E; BBS; MAS
Ye (2020)	41/41	(5.42±1.03)/(5.53±1.11)	RAGT+ conventional rehabilitation	Conventional rehabilitation	30min/day, 5days/week	3 months	–	LokoHelp	BBS; MAS
Druzbicki (2013)	26/9	(10.5±2.2)/(11.0±2.3)	RAGT+ conventional rehabilitation	Conventional rehabilitation	45min/day	20 times RAGT	–	Lokomat	Walking speed
Wallard (2018)	14/16	(8.3±1.2)/(9.6±1.7)	RAGT+ conventional rehabilitation	Conventional rehabilitation	30min/day, 5days/week	4 weeks	–	Lokomat	GMFM-88 D, E; Walking speed
Klobucká (2020)	21/26	(18.3±3.84)/(23.4±5.33)	RAGT+ conventional rehabilitation	Conventional rehabilitation	30min/day, 4–5days/week	20 times RAGT	3–4 months	Lokomat	GMFM-88
Ma (2021)	15/15	(8.27±2.74)/(9.82±2.68)	RAGT+ conventional rehabilitation	Conventional rehabilitation	30min/day, 5days/week	8 weeks	1 month	Lokomat	GMFM-88 D, E; 6MWT
Zheng (2021)	35/35	(5.16±0.53)/(5.24±0.59)	RAGT+ conventional rehabilitation	Conventional rehabilitation	30min/day	3 months	–	Lokomat	GMFM-88 D, E; BBS
Wu (2017)	11/10	(11.3±3.8)/(10.5±2.6)	RAGT+ conventional rehabilitation	Conventional rehabilitation	30-40min/day, 3days/week	6 weeks	2 months	3DCaLT	GMFM-88; MAS
Smania (2011)	9/9	(13±2.83)/(12±3.08)	RAGT+ conventional rehabilitation	Conventional rehabilitation	40min/day, 5days/week	2 weeks	1 month	Gait Trainer I	6MWT; Walking speed
Yasar (2021)	13/13	(10.46±2.76)/(9.69±2.32)	RAGT+ conventional rehabilitation	Conventional rehabilitation	25min/day, 2days/week	8 weeks	–	–	MAS

*E* experimental group, *C* control group

### Quality and risk of bias assessment

**Fig. 2:**
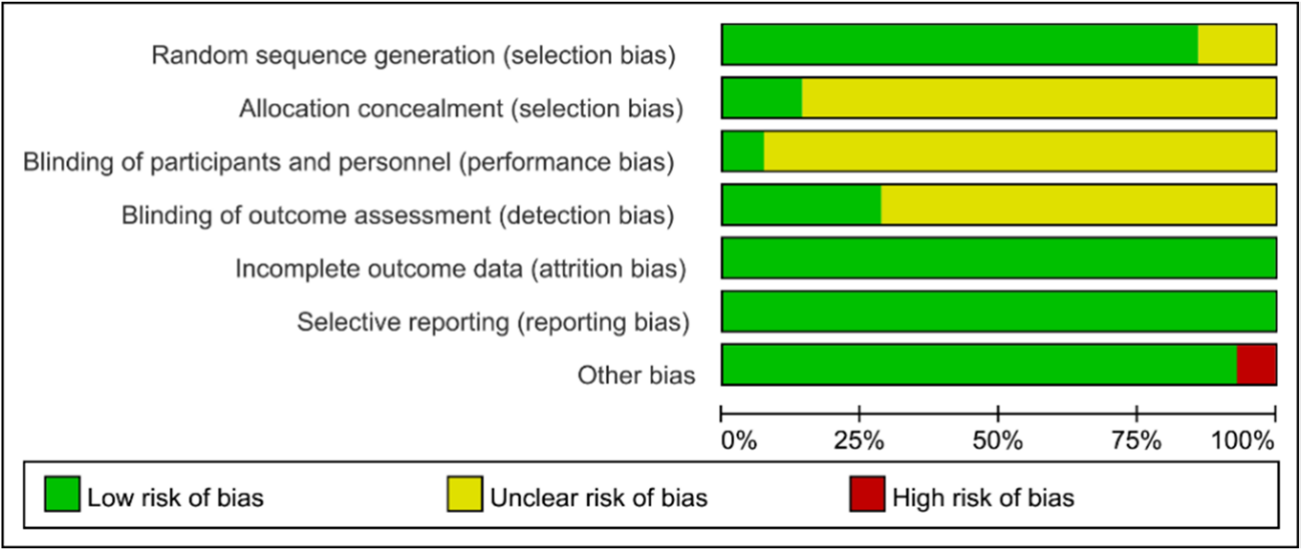
Risk assessment of bias

All studies mentioned “random” or “randomized grouping,” but two studies [21, 23] did not specify the specific randomized scheme. Two studies [25, 27] used opaque envelopes to complete allocation hiding, while the remaining studies did not mention allocation sequence hiding. One study [24] blinded the treatment, and four studies [23–25, 28] were blinded to outcome assessors. The data results included in the literature were complete and not reported selectively. One study [26] had another bias. Nine included studies [16, 18, 19, 23–28] were of high quality, and five [15, 17, 20–22] were of moderate quality, with a mean score of 7.14. The results of the quality assessment of the included studies are shown in Fig. 2 and Table 2.

**Table 2:** Physiotherapy evidence database scores of the included studies

Study	1	2	3	4	5	6	7	8	9	10	11	Scores	Quality level
Jin (2012)	Yes	1	0	1	0	0	0	1	1	1	1	7/10	Medium
Zhu (2016)	Yes	1	0	1	1	0	0	1	1	1	1	7/10	High
Lv (2017)	Yes	1	0	1	0	0	1	1	1	1	1	7/10	High
Yin (2017)	Yes	1	0	1	0	0	0	1	1	1	1	6/10	Medium
Zhang (2018)	Yes	1	0	1	0	0	1	1	1	1	1	7/10	High
Ye (2020)	Yes	1	0	1	0	0	0	1	1	1	1	6/10	Medium
Druzbicki (2013)	Yes	1	0	1	0	0	1	1	1	1	1	7/10	High
Wallard (2018)	Yes	1	0	1	0	0	1	1	1	1	1	7/10	High
Klobucká (2020)	Yes	1	0	1	1	1	1	1	1	1	1	9/10	High
Ma (2021)	Yes	1	0	1	0	0	0	1	1	1	1	7/10	Medium
Zheng (2021)	Yes	1	0	1	0	0	0	1	1	1	1	6/10	Medium
Wu (2017)	Yes	1	1	1	0	0	0	1	1	1	1	7/10	High
Smania (2011)	Yes	1	1	1	1	1	1	1	1	1	1	10/10	High
Yasar (2021)	Yes	1	0	1	0	0	1	1	1	1	1	7/10	High

### Meta-analysis

#### GMFM-88 D score

**Fig. 3:**
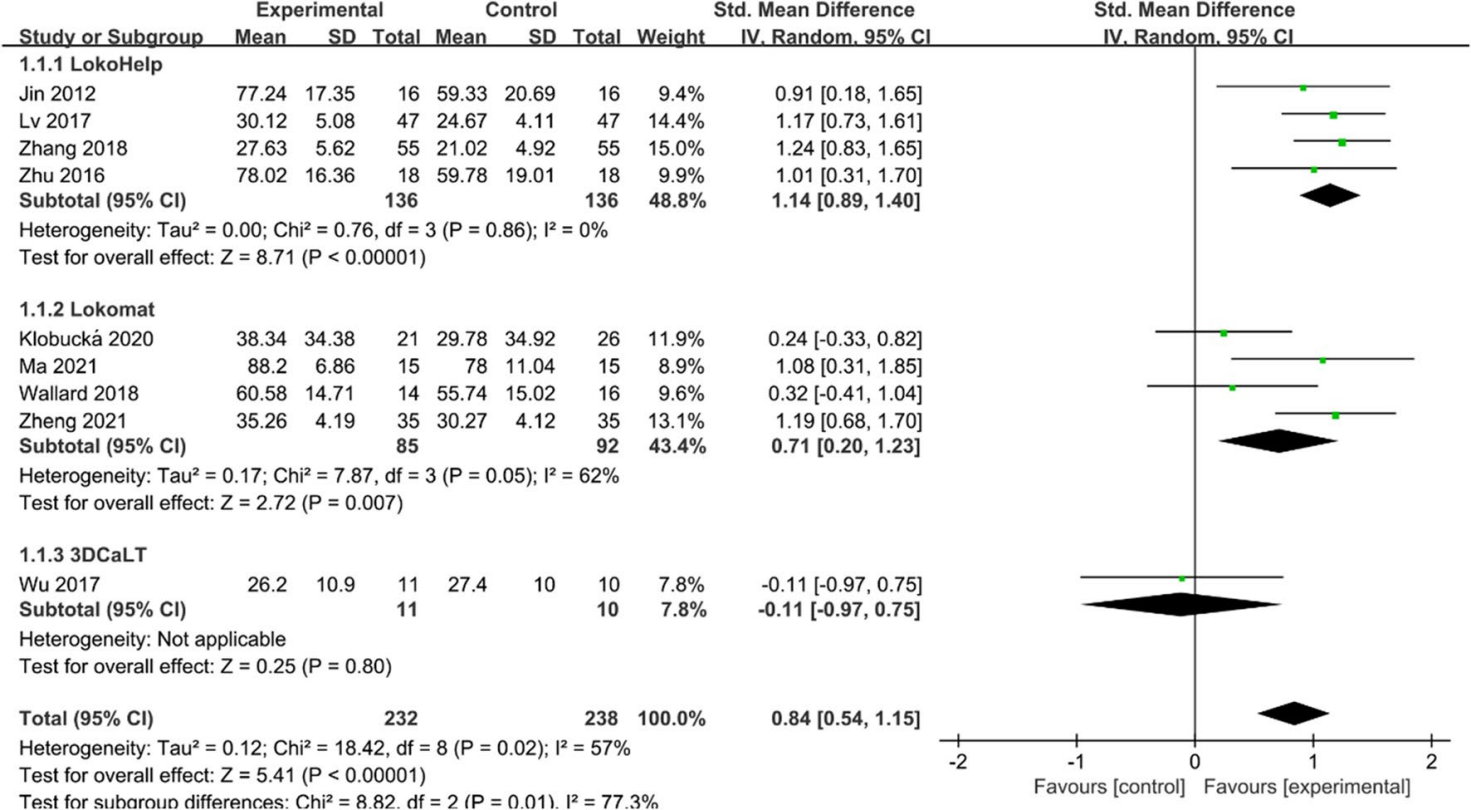
Effects of RAGT on GMFM-88 D scores of patients with CP

Nine studies [16, 18–22, 24, 26, 27] reported GMFM-88 D scores. Heterogeneity tests suggested significant heterogeneity among studies (*I*^2^ = 57%, *P* = 0.02), and a random effect model was used to combine effect sizes. Overall, the GMFM-88 D score was higher in the experimental group than in the control group (SMD = 0.84, 95% CI 0.54 to 1.15, *P* < 0.05) (Fig. 3). Subgroup analysis showed that GMFM-88 D scores were significantly higher in both the LokoHelp and Lokomat groups than in the control group (SMD = 1.14, 95% CI 0.89 to 1.40, *P* < 0.05 and SMD = 0.71, 95% CI 0.20 to 1.23, *P* < 0.05). However, the difference between the 3DCaLT and control groups was insignificant (SMD = −0.11, 95% CI −0.97 to 0.75, *P* > 0.05).

#### GMFM-88 E score

**Fig. 4:**
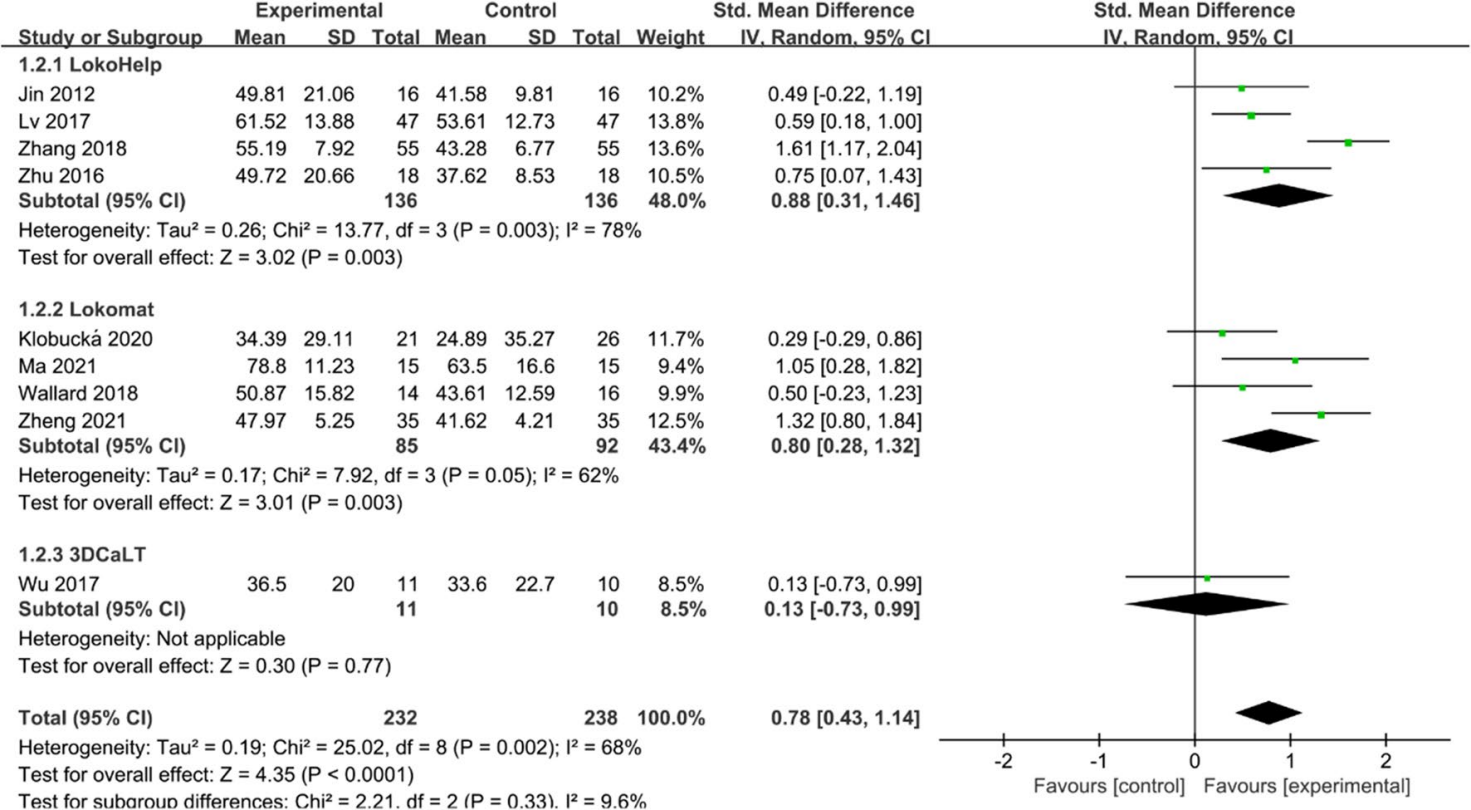
Effects of RAGT on GMFM-88 E scores of patients with CP

Nine studies [16, 18–22, 24, 26, 27] reported GMFM-88 E scores. Heterogeneity tests suggested significant heterogeneity among studies (*I*^2^ = 68%, *P* = 0.002), and a random effect model was used to combine effect sizes. Overall, GMFM-88 E scores were significantly higher in the experimental group than in the control group (SMD = 0.78, 95% CI 0.43 to 1.14, *P* < 0.05) (Fig. 4). Subgroup analysis showed that GMFM-88 E scores were higher in both the LokoHelp and Lokomat groups than in the control group (SMD = 0.88, 95% CI 0.31 to 1.46, *P* < 0.05 and SMD = 0.80, 95% CI 0.28 to 1.32, *P* < 0.05). However, there was no significant difference between the 3DCaLT and control groups (SMD = 0.13, 95% CI −0.73 to 0.99, *P* > 0.05).

#### BBS

**Fig. 5:**
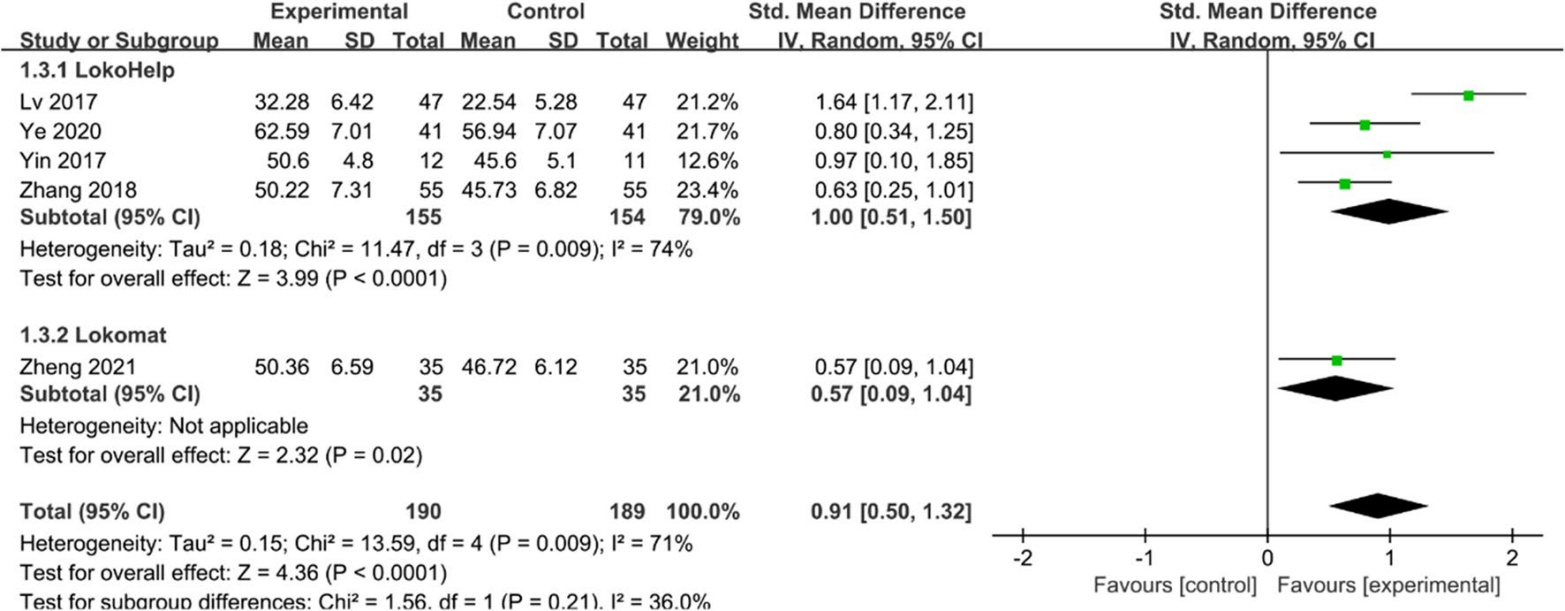
Effects of RAGT on BBS scores of patients with CP

Five studies [15–18, 22] reported BBS scores. Heterogeneity tests suggested significant heterogeneity among studies (*I*^2^ = 71%, *P* = 0.009), and a random effect model was used to combine effect sizes. Overall, BBS scores were higher in the experimental group than in the control group (SMD = 0.91, 95% CI 0.50 to 1.32, *P* < 0.05) (Fig. 5). Subgroup analysis showed that BBS scores were higher in both the LokoHelp and Lokomat groups than in the control group (SMD = 1.00, 95% CI 0.51 to 1.50, *P* < 0.05 and SMD = 0.57, 95% CI 0.09 to 1.04, *P* < 0.05).

#### 6MWT

**Fig. 6:**
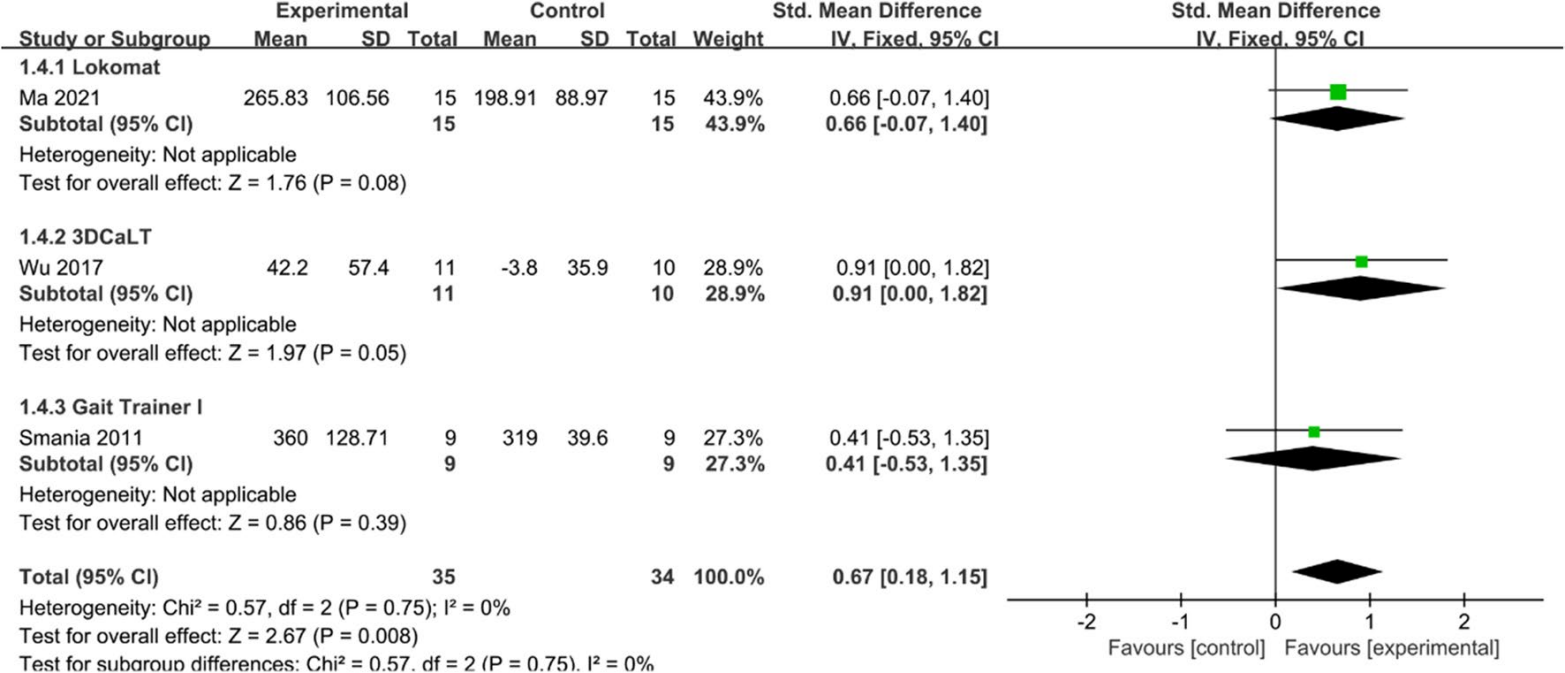
Effects of RAGT on 6MWT of patients with CP

Three studies [21, 25, 27] reported the results of the 6MWT evaluation. Heterogeneity tests suggested no significant heterogeneity among studies (*I*^2^ = 0%, *P* = 0.75), and a fixed effect model was used to combine effect sizes. Overall, the results of the 6MWT evaluation were higher in the experimental group than in the control group (SMD = 0.67, 95% CI 0.18 to 1.15, *P* < 0.05) (Fig. 6). Subgroup analysis showed that 6MWT evaluation results were higher in the 3DCaLT group than in the control group (SMD = 0.91, 95% CI 0.00 to 1.82, *P* = 0.05). 6MWT evaluation results in both the Lokomat and Gait Trainer 1 group were not statistically significant compared to the control group (SMD = 0.66, 95% CI −0.07 to 1.40, *P* > 0.05 and SMD = 0.41, 95% CI −0.53 to 1.35, *P* > 0.05).

#### Walking speed

**Fig. 7:**
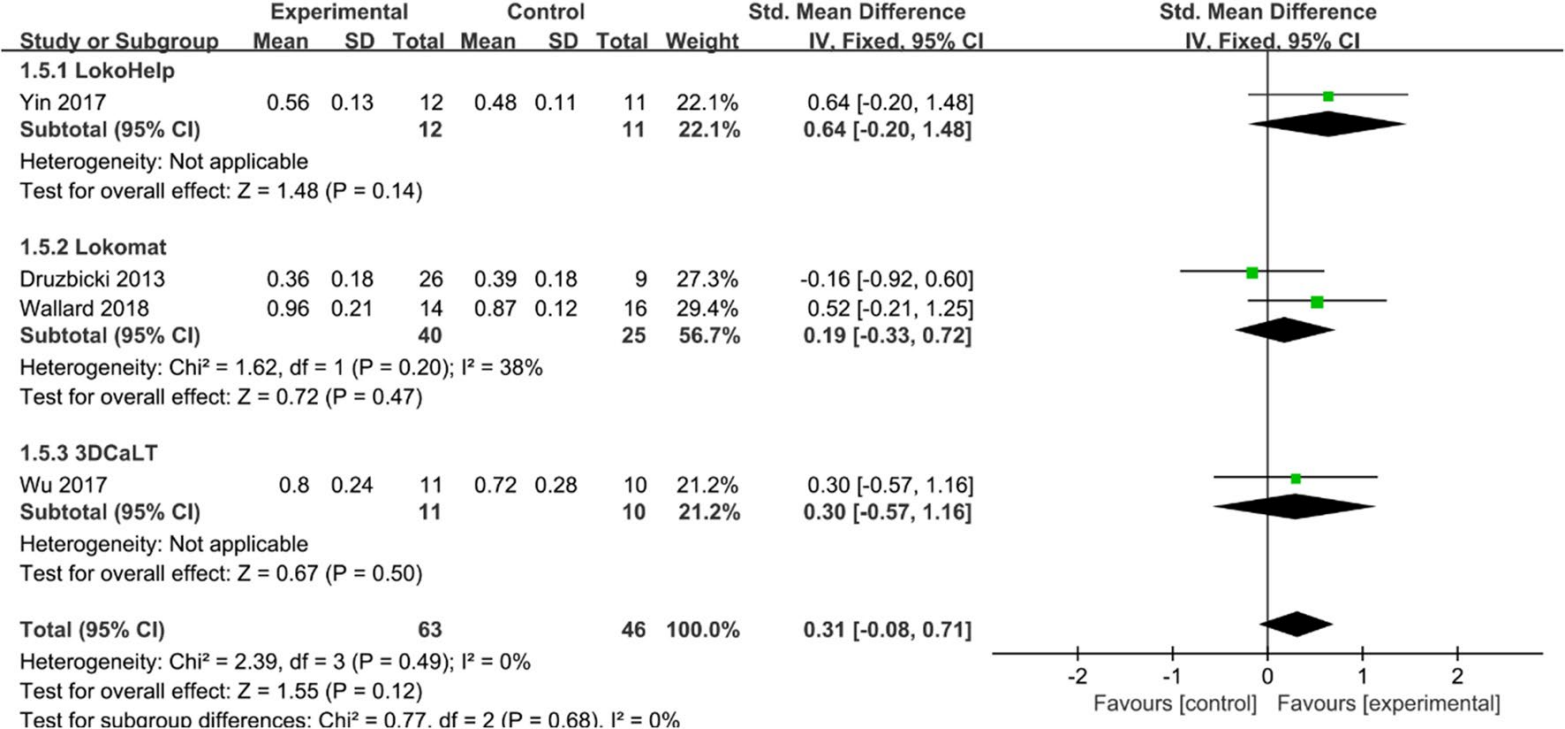
Effects of RAGT on the walking speed of patients with CP

Four studies [17, 23, 26, 27] reported walking speed (m/s) in patients with CP after treatment. Heterogeneity tests suggested no significant heterogeneity among studies (*I*^2^ = 0%, *P* = 0.49), and a fixed effect model was used to combine effect sizes. Overall, the walking speed of patients in the experimental group was higher than that of the control group. The difference between the experimental and control groups was not statistically significant (SMD = 0.31, 95% CI −0.08 to 0.71, *P* > 0.05) (Fig. 7).

#### MAS

**Fig. 8:**
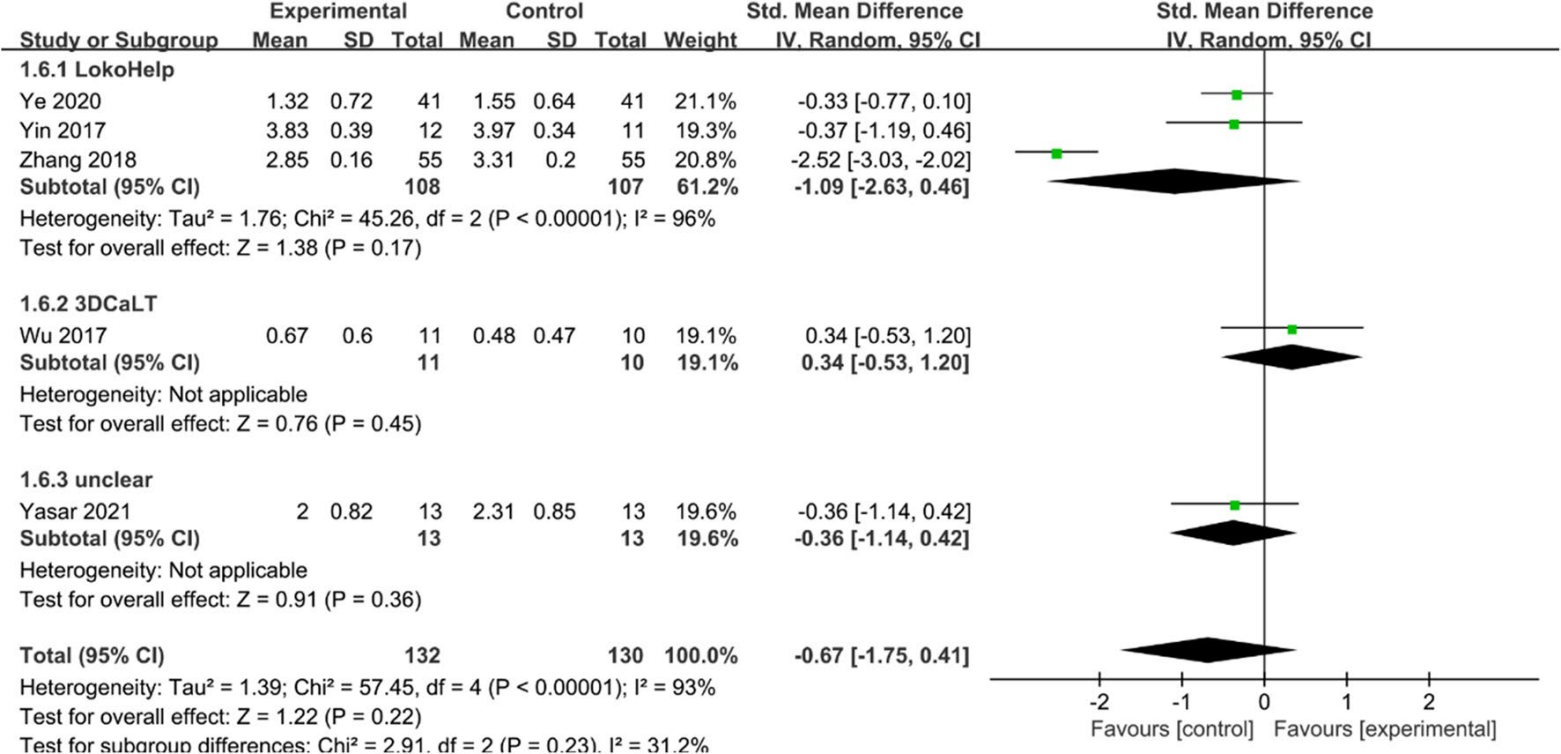
Effects of RAGT on MAS scores of patients with CP

Five studies [15, 17, 18, 27, 28] reported MAS scores. Heterogeneity tests suggested significant heterogeneity among studies (*I*^2^ = 93%, *P* < 0.00001), and a random effect model was used to combine effect sizes. Overall, the MAS scores were lower in the experimental group than in the control group. The difference between the experimental and control groups was not statistically significant (SMD = −0.67, 95% CI −1.75 to 0.41, *P* > 0.05) (Fig. 8).

### Network meta-analysis

#### Evidence network

**Fig. 9:**
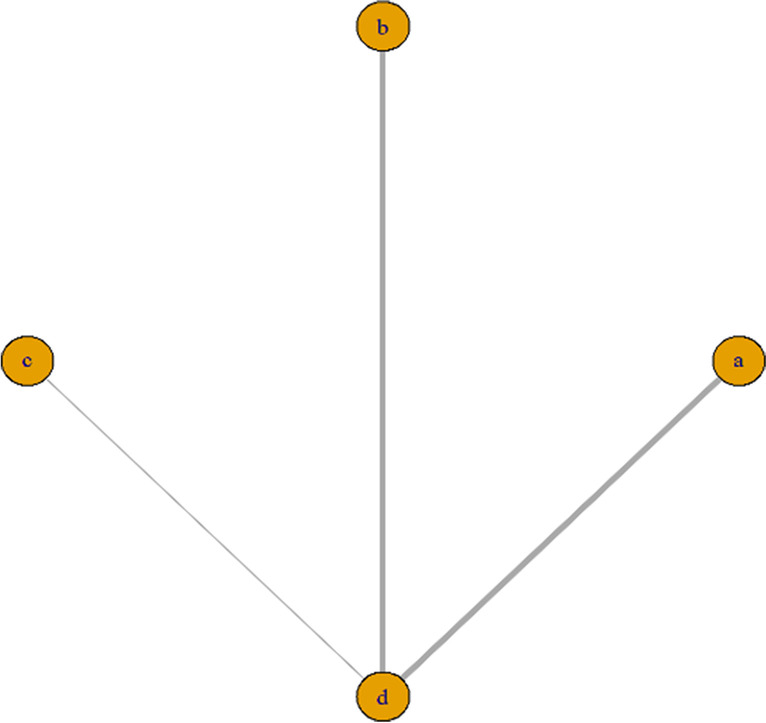
The network relationship of efficacy Comparison of different robot

The GMFM-88 E score, more closely related to lower limb motor function, was used to compare the efficacy of different robotic. Of the studies reporting GMFM-88 E scores, four studies [16, 18–20] used the LokoHelp rehabilitation robot (RECK Company), four studies [21, 22, 24, 26] used the Lokomat children's version rehabilitation robot (HOCOMA Company), and one study [27] used 3DCaLT robot device. The network relationship is shown in Fig. 9 (a, LokoHelp; b, Lokomat; c, 3DCaLT; d, control).

#### Consistency test

There was no closure of the loop between interventions in this study. Therefore, consistency testing was not required.

#### Convergence diagnosis

**Fig. 10:**
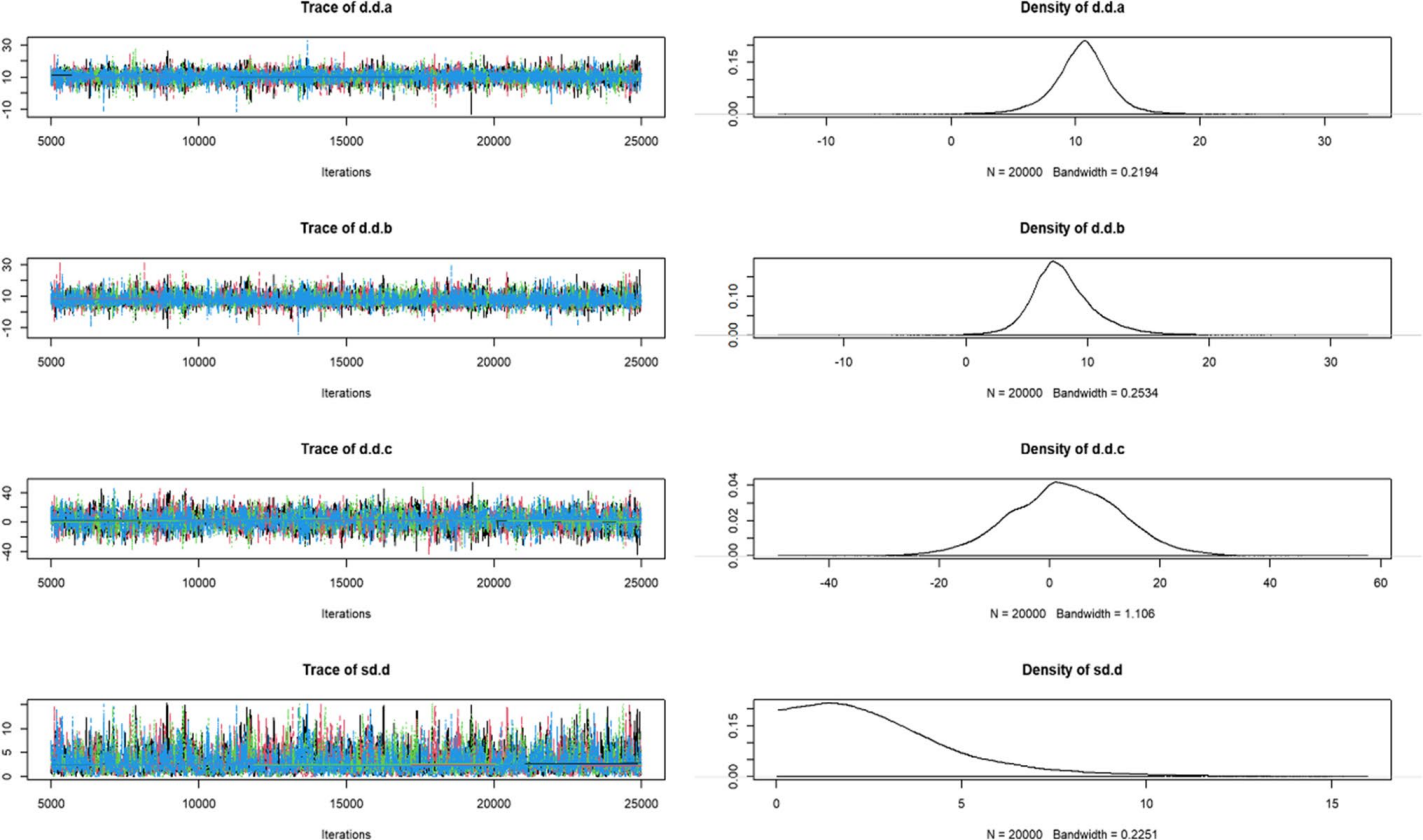
Track density map of the GMFM-88 E score

A convergence diagnosis of the included studies revealed bandwidth values close to 0, suggesting good convergence (Fig. 10).

#### Probability ranking

**Fig. 11:**
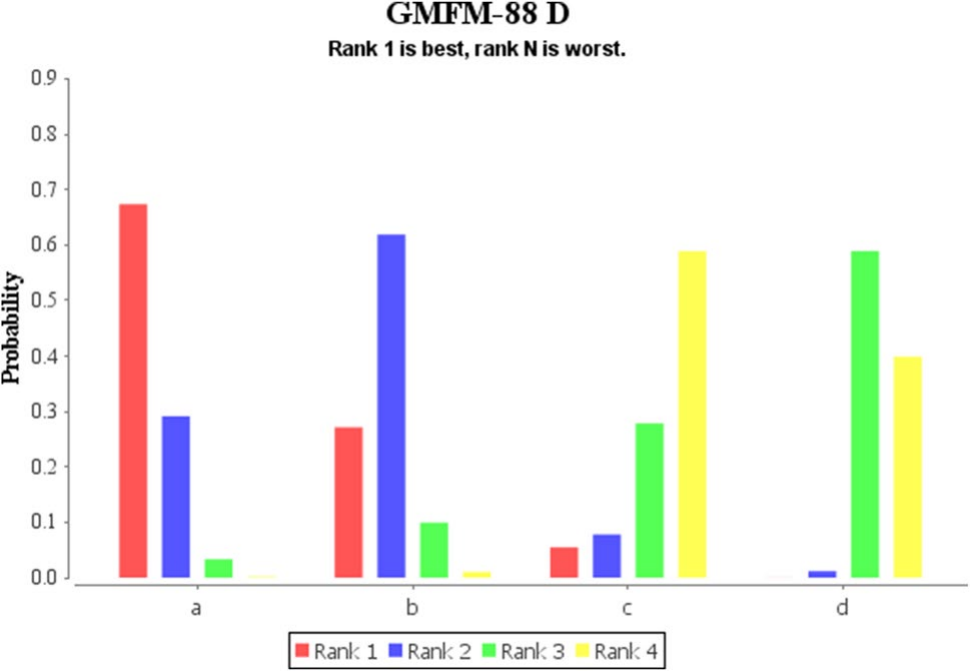
Rank of GMFM-88 D scores

**Fig. 12:**
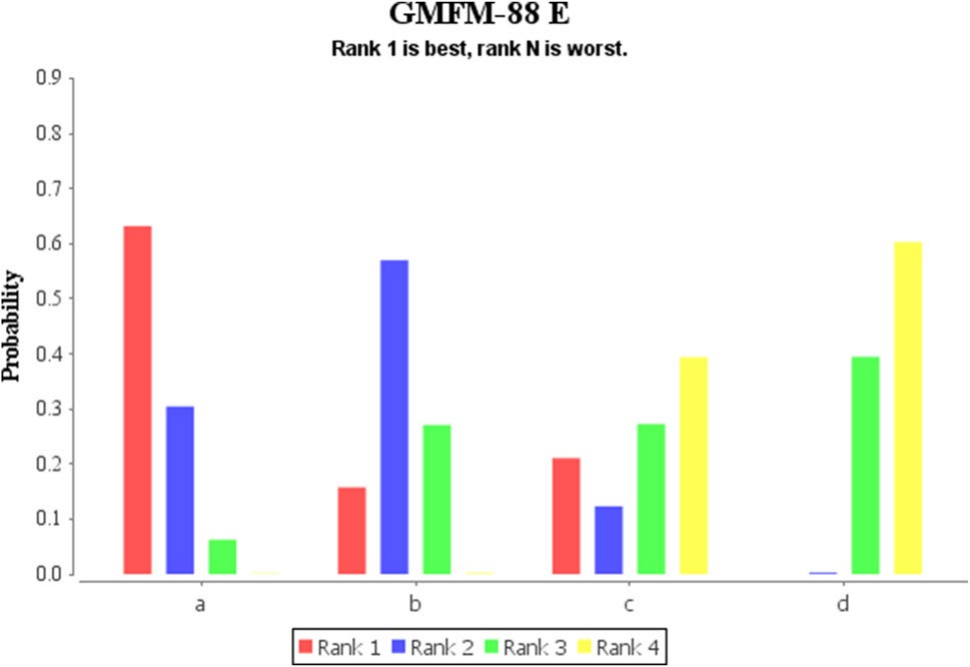
Rank of GMFM-88 E scores

The network meta-analysis revealed that the best ranking of probabilities for the effect of 3 different robots on GMFM-88 D scores was LokoHelp (*P* = 0.66) > Lokomat (*P* = 0.28) > 3DCaLT (*P* = 0.06) and the best ranking of probabilities for the GMFM-88 E score was LokoHelp (*P* = 0.63) > 3DCaLT (*P* = 0.21) > Lokomat (*P* = 0.16) (Fig. 11, Fig. 12, Table 3).

**Table 3:** Best probability ranking

Interventions	LokoHelp	Lokomat	3DCaLT	Control
GMFM-88 D	0.66	0.28	0.06	0.00
GMFM-88 E	0.63	0.16	0.21	0.00

### Adverse reaction

One study [17] reported that RAGT training caused a small number of patients to experience mild discomfort from rubbing the skin on the inner root of the thigh from wearing the robot device, which was relieved by rest and did not interfere with the next day’s treatment. No adverse effects were reported in other studies.

### Subgroup analysis of primary outcomes

GMFM-88 D and E scores were analyzed in subgroups according to the intervention time and patient age. The analysis results generally follow the above analysis (Table 4).

**Table 4:** Subgroup analysis of RAGT on patients with CP

Subgroup analysis		Studies	SMD (95% CI)	*P*	*X*^2^	*I*^2^ (%)	Tau^2^
GMFM-88 D							
Intervention length (Fixed)	≤6 weeks	3	0.19 [−0.21, 0.59]	0.35	0.62	0%	–
	8 weeks	4	1.12 [0.83, 1.42]	<0.0001	0.76	0%	–
	3 months	2	1.18 [0.84, 1.51]	<0.0001	0	0%	–
Age (Fixed)	≤10 years	6	1.08 [0.86, 1.31]	<0.0001	5.42	8%	–
	>10 years	2	0.13 [−0.35, 0.61]	0.59	0.45	0%	–
GMFM-88 E							
Intervention length (Random)	≤6 weeks	3	0.32 [−0.08, 0.72]	0.12	0.43	0%	0
	8 weeks	4	1.02 [0.47, 1.57]	0.0003	9.07	67%	0.21
	3 months	2	0.94 [0.22, 1.65]	0.01	4.65	78%	0.21
Age (Random)	≤10 years	6	0.95 [0.53, 1.38]	<0.0001	16.66	70%	0.19
	>10 years	2	0.24 [−0.24, 0.72]	0.33	0.09	0%	0

### Sensitivity analysis

The meta-analysis results were analyzed for sensitivity using a one-by-one exclusion method, removing one study at a time. The results showed no significant change from the above results, indicating that the meta-analysis results were relatively stable.

### Publication bias

**Fig. 13:**
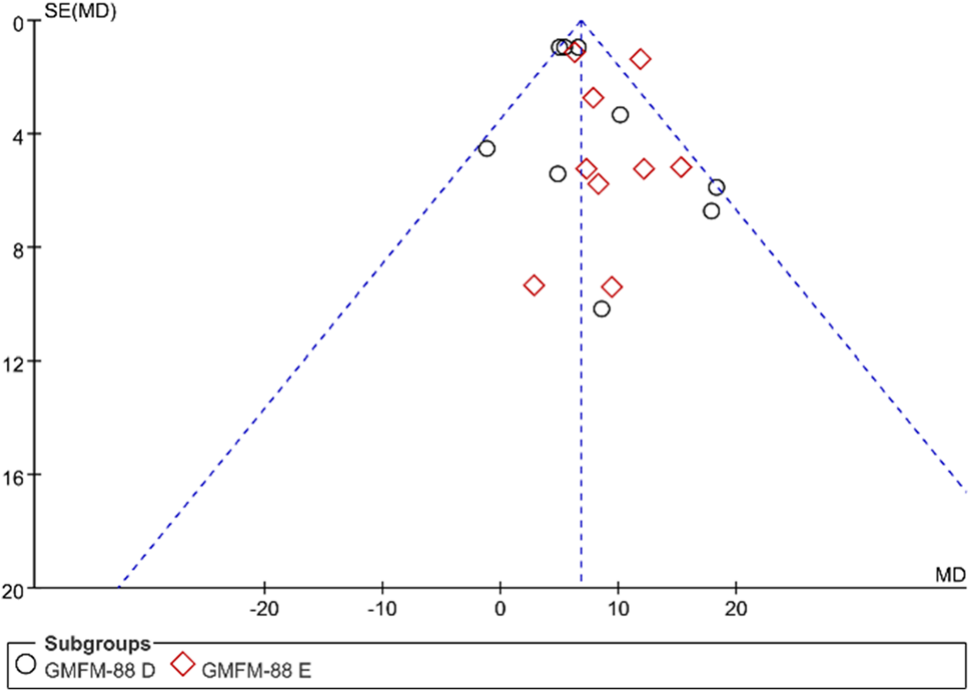
Funnel plot

No significant asymmetry was seen in the funnel plot of the study with GMFM-88 D and E scores as outcome indicators. It is suggested that the results of data analysis are less influenced by publication bias (Fig. 13).

## Discussion

Lower limb motor dysfunction is one of the most common symptoms in patients with CP [29, 30], leading to an inability to walk or abnormal gait patterns, such as horseshoe foot or crouching gait [31]. It can cause secondary injuries and have a severe impact on the quality of life of patients. Therefore, the balance of lower limbs and walking functions are essential in rehabilitating patients with CP [32, 33]. RAGT, as an emerging rehabilitation therapy, brings new hope for the functional rehabilitation of CP patients [34]. This study analyzed the literature on RAGT for lower limb motor function in patients with CP using a network meta-analysis. We compared the clinical efficacy of different robotic devices.

The meta-analysis showed that RAGT training improved GMFM-88 D and E area scores, BBS scores, and 6MWT significantly better than conventional rehabilitation in CP patients. However, the effect of improved gait speed and lower limb muscle spasticity is unclear. The network meta-analysis results showed that RAGT improved lower limb motor function in CP patients, with LokoHelp most likely to improve lower limb motor function to a greater extent in CP patients.

Central nervous system plasticity theory and motor learning theory, closely related to the reconstruction of brain cell function, suggest that repetitive movements can induce plastic changes in the brain. Neural plasticity changes are primarily realized by rebuilding neural networks [35–38]. The therapeutic mechanism of RAGT on lower limb motor function in patients with CP may be related to the improvement of nervous system plasticity [39]. Shin showed that repetitive RAGT training promotes functional network reorganization in the brain’s motor cortex and stimulates neuroplasticity [40]. Youssofzadeh found that RAGT training significantly improved functional network connectivity around the sensory-motor areas of the brain and enhanced functional connectivity between the prefrontal and parietal lobes after EEG analysis in healthy subjects [41].

The following limitations exist in this study: the total number of subjects included in the literature was small; some of the literature was not allocation sequence concealed and blinded, which may lead to a potential risk of bias; there were age differences in the study population and varying severity of the condition, which may have had an impact on the rehabilitation outcome; the frequency and periodicity of interventions varied in the literature, which may have biased the findings; some of the literature had short treatment periods.

## Conclusion

In conclusion, RAGT training improved the motor function of the lower limbs of CP patients, with LokoHelp probably having the best treatment effect. This positively impacted the patient’s quality of life, balance function, and motor control. However, the evidence for the efficacy of RAGT in improving gait speed and muscle spasticity is insufficient and needs further exploration. In addition, the small sample size of the literature included in this study and the variable quality of the articles may have impacted the analysis results. Therefore, adequate sample size and high-quality and long-term follow-up studies are still needed to explore the clinical efficacy of RAGT in depth.

## Data Availability

The data used to support the findings of this study are available from the corresponding author upon request.

## References

[1] Graham HK, Rosenbaum P, Paneth N, Dan B, Lin JP, Damiano DL, Becher JG, Gaebler-Spira D, Colver A, Reddihough DS (2016). Cerebral palsy. Nat Rev Dis Primers.

[2] Sadowska M, Sarecka-Hujar B, Kopyta I (2020). Cerebral palsy: current opinions on definition, epidemiology, risk factors, classification and treatment options. Neuropsychiatr Dis Treat.

[3] Schwabe AL (2020). Comprehensive care in cerebral palsy. Phys Med Rehabil Clin N Am.

[4] Subspecialty Group of Rehabilitation, the Society of Pediatrics, Chinese Medical Association (2020). Rehabilitation strategy and recommendation for motor dysfunction in children with cerebral palsy. Chin J Pediatr.

[5] Korzeniewski SJ, Slaughter J, Lenski M, Haak P, Paneth N (2018). The complex aetiology of cerebral palsy. Nat Rev Neurol.

[6] Xiong HC, Chen JH, Wang J, Zhu DN, Xiao N, Zhou YP, Zhou ZH, Tang GH, Yang YH (2021). Effects of robot-assisted gait training on the gross motor and balance function of children with spastic cerebral palsy. Zhengzhou University (Medical Sciences).

[7] Peri E, Turconi AC, Biffi E, Maghini C, Panzeri D, Morganti R, Pedrocchi A, Gagliardi C (2017). Effects of dose and duration of robot-assisted gait training on walking ability of children affected by cerebral palsy. Technol Health Care.

[8] Ammann-Reiffer C, Bastiaenen CHG, Meyer-Heim AD, van Hedel HJA (2020). Lessons learned from conducting a pragmatic, randomized, crossover trial on robot-assisted gait training in children with cerebral palsy (PeLoGAIT). J Pediatr Rehabil Med.

[9] Jin LH, Yang SS, Choi JY, Sohn MK (2020). The effect of robot-assisted gait training on locomotor function and functional capability for daily activities in children with cerebral palsy: a single-blinded, randomized cross-over trial. Brain Sci.

[10] Lerner ZF, Damiano DL, Park HS, Gravunder AJ, Bulea TC (2017). A robotic exoskeleton for treatment of crouch gait in children with cerebral palsy: design and initial application. IEEE Trans Neural Syst Rehabil Eng.

[11] Borggraefe I, Schaefer JS, Klaiber M, Dabrowski E, Ammann-Reiffer C, Knecht B, Berweck S, Heinen F, Meyer-Heim A (2010). Robotic-assisted treadmill therapy improves walking and standing performance in children and adolescents with cerebral palsy. Eur J Paediatr Neurol.

[12] Digiacomo F, Tamburin S, Tebaldi S, Pezzani M, Tagliafierro M, Casale R, Bartolo M (2019). Improvement of motor performance in children with cerebral palsy treated with exoskeleton robotic training: a retrospective explorative analysis. Restor Neurol Neurosci.

[13] Lobato Garcia L, González González Y, Da Cuña Carrera I, Alonso Calvete A (2020). Benefits of robotics in gait rehabilitation in cerebral palsy: a systematic review. Rehabilitacion (Madr).

[14] Guo HB, Zhou X, Du Q (2021). Research progress of lower limb rehabilitation robot in improving the walking ability of children with cerebral palsy. Chin J Rehabil.

[15] Ye N, Fan QH, Chen ZH, Wu TT, Shen LF (2020). Influence study of lower limb robot-assisted gait training on rehabilitation effects and quality of life of children patients with cerebral palsy. Chin Med Equip.

[16] Lv N, Shang Q, Ma CY, Li JJ, Zhang QM (2017). Rehabilitation effect of rehabilitation robot on children with spastic cerebral palsy. Chin J Pract Nerv Dis.

[17] Yin ZL, Meng ZX, Xue YJ, Ren SW, Jin X (2017). Effects of rehabilitation robot-assisted walking training on walking ability in adult patients with cerebral palsy. Chin J Rehabil Med.

[18] Zhang RJ (2018). Rehabilitation robot-assisted walking training on lower limb muscle tone in children with cerebral palsy. Chin J Pract Med.

[19] Zhu MJ (2016). Observation of curative effect by rehabilitation robot in rehabilitation therapy for cerebral palsy children. Chin Pract Med.

[20] Jin X, Meng ZX, Yin ZL, Zhang XB, Wang JB, Fan ZL, Chen B, Ke MH (2012). The effects of robot-aided walking training on the walking ability in children with cerebral palsy. Chin J Rehabil Med.

[21] Ma TT, Zhang H (2021). Effect of robotic-assisted gait training on motor and walking for children with spastic cerebral palsy. Chin J Rehabil Theory Pract.

[22] Zheng HC, Wang Q, Yang L (2021). Effect of lower limb robot combined with exercise thera. Clin Res Pract.

[23] Drużbicki M, Rusek W, Snela S, Dudek J, Szczepanik M, Zak E, Durmala J, Czernuszenko A, Bonikowski M, Sobota G (2013). Functional effects of robotic-assisted locomotor treadmill thearapy in children with cerebral palsy. J Rehabil Med.

[24] Klobucká S, Klobucký R, Kollár B (2020). Effect of robot-assisted gait training on motor functions in adolescent and young adult patients with bilateral spastic cerebral palsy: a randomized controlled trial. Neurorehabil.

[25] Smania N, Bonetti P, Gandolfi M, Cosentino A, Waldner A, Hesse S, Werner C, Bisoffi G, Geroin C, Munari D (2011). Improved gait after repetitive locomotor training in children with cerebral palsy. Am J Phys Med Rehabil.

[26] Wallard L, Dietrich G, Kerlirzin Y, Bredin J (2018). Effect of robotic-assisted gait rehabilitation on dynamic equilibrium control in the gait of children with cerebral palsy. Gait Posture.

[27] Wu M, Kim J, Arora P, Gaebler-Spira DJ, Zhang Y (2017). Effects of the integration of dynamic weight shifting training into treadmill training on walking function of children with cerebral palsy: a randomized controlled study. Am J Phys Med Rehabil.

[28] Yaşar B, Atıcı E, Razaei DA, Saldıran TÇ (2021). Effectiveness of robot-assisted gait training on functional skills in children with cerebral palsy. J Pediatr Neurol.

[29] Bartels EM, Korbo L, Harrison AP (2020). Novel insights into cerebral palsy. J Muscle Res Cell Motil.

[30] Wu M, Kim J, Gaebler-Spira DJ, Schmit BD, Arora P (2017). Robotic resistance treadmill training improves locomotor function in children with cerebral palsy: a randomized controlled pilot study. Arch Phys Med Rehabil.

[31] Goldstein M, Harper DC (2001). Management of cerebral palsy: equinus gait. Dev Med Child Neurol.

[32] Roostaei M, Akbarfahimi N, Dalvand H, Abedi S (2021). The relationship between functional motor status and self-evaluation in individuals with cerebral palsy: a systematic review. Iran J Child Neurol.

[33] Wilson JL, Kim YM, O'Malley JA, Gelineau-Morel R, Gilbert L, Bain JM, Aravamuthan BR (2022). Cerebral palsy in child neurology and neurodevelopmental disabilities training: an unmet need. J Child Neurol.

[34] van Hedel HJA, Severini G, Scarton A, O'Brien A, Reed T, Gaebler-Spira D, Egan T, Meyer-Heim A, Graser J, Chua K (2018). Advanced Robotic Therapy Integrated Centers (ARTIC): an international collaboration facilitating the application of rehabilitation technologies. J Neuroeng Rehabil.

[35] Wiart L, Rosychuk RJ, Wright FV (2016). Evaluation of the effectiveness of robotic gait training and gait-focused physical therapy programs for children and youth with cerebral palsy: a mixed methods RCT. BMC Neurol.

[36] Dayan E, Cohen LG (2011). Neuroplasticity subserving motor skill learning. Neuron.

[37] Xie ZC, Tang JK (2006). Analysis of training techniques for the functional rehabilitation of cerebral palsy. Chin J Clin Rehabil.

[38] Li L, Huang H, Jia Y, Yu Y, Liu Z, Shi X, Wang F (2021). Systematic review and network meta-analysis of noninvasive brain stimulation on dysphagia after stroke. Neural Plast.

[39] de Oliveira RMW (2020). Neuroplasticity. J Chem Neuroanat.

[40] Shin J, Yang S, Park C, Lee Y, You SJH (2022). Comparative effects of passive and active mode robot-assisted gait training on brain and muscular activities in sub-acute and chronic stroke. Neurorehabil.

[41] Youssofzadeh V, Zanotto D, Wong-Lin K, Agrawal SK, Prasad G (2016). Directed functional connectivity in fronto-centroparietal circuit correlates with motor adaptation in gait training. IEEE Trans Neural Syst Rehabil Eng.

